# Structural impairments in hippocampal and occipitotemporal networks specifically contribute to decline in place and face category processing but not to other visual object categories in healthy aging

**DOI:** 10.1002/brb3.2127

**Published:** 2021-06-29

**Authors:** José Bourbon‐Teles, Lília Jorge, Nádia Canário, Miguel Castelo‐Branco

**Affiliations:** ^1^ Coimbra Institute for Biomedical Imaging and Translational Research (CIBIT) Institute for Nuclear Sciences Applied to Health (ICNAS) University of Coimbra Coimbra Portugal; ^2^ Faculty of Medicine University of Coimbra Coimbra Portugal

**Keywords:** fornix, Healthy aging, hippocampus, inferior longitudinal fasciculus, place processing, white matter

## Abstract

**Background:**

Functional neuroimaging studies have identified a set of nodes in the occipital–temporal cortex that preferentially respond to faces in comparison with other visual objects. By contrast, the processing of places seems to rely on parahippocampal cortex and structures heavily implicated in memory (e.g., the hippocampus). It has been suggested that human aging leads to decreased neural specialization of core face and place processing areas and impairments in face and place perception.

**Methods:**

Using mediation analysis, we tested the potential contribution of micro‐ and macrostructure within the hippocampal and occipitotemporal systems to age‐associated effects in face and place category processing (as measured by 1‐back working memory tasks) in 55 healthy adults (age range 23–79 years). To test for specific contributions of the studied structures to face/place processing, we also studied a distinct tract (i.e., the anterior thalamic radiation [ATR]) and cognitive performance for other visual object categories (objects, bodies, and verbal material). Constrained spherical deconvolution‐based tractography was used to reconstruct the fornix, the inferior longitudinal fasciculus (ILF), and the ATR. Hippocampal volumetric measures were segmented from FSL‐FIRST toolbox.

**Results:**

It was found that age associates with (a) decreases in fractional anisotropy (FA) in the fornix, in right ILF (but not left ILF), and in the ATR (b) reduced volume in the right and left hippocampus and (c) decline in visual object category processing. Importantly, mediation analysis showed that micro‐ and macrostructural impairments in the fornix and right hippocampus, respectively, associated with age‐dependent decline in place processing. Alternatively, microstructural impairments in right hemispheric ILF associated with age‐dependent decline in face processing. There were no other mediator effects of micro‐ and macrostructural variables on age–cognition relationships.

**Conclusion:**

Together, the findings support specific contributions of the fornix and right hippocampus in visuospatial scene processing and of the long‐range right hemispheric occipitotemporal network in face category processing.

## Graphical abstract

Micro‐ and macrostructural impairments in the fornix and right hippocampus are associated with age‐dependent decline in place processing. Conversely, microstructural changes in right hemispheric inferior longitudinal fasciculus are associated with age‐dependent decline in face processing.

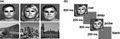

## 1 INTRODUCTION

Understanding how aging affects visual perception of distinct object/scene categories remains a subject of active research. Much has been studied regarding the neural underpinnings supporting human visual object processing and recognition.

Functional neuroimaging studies are in support of a subset of cortical nodes located in the occipitotemporal cortex (particularly in the right hemisphere) which typically respond to faces in comparison with other visual objects and include the fusiform face area (FFA), perirhinal cortex (PrC), occipital face area (OFA), and the superior temporal sulcus (STS) (Barense et al., 2010; Behrmann & Plaut, 2013; Canário et al., 2016; Direito et al., 2019; Grill‐Spector, 2003; Grill‐Spector et al., 2004, 2017; Mundy et al., 2013; Pitcher et al., 2011; Rebola & Castelo‐Branco, 2014). Alternatively, the processing of places/scenes seems to rely on neural substrates largely implicated in memory such as the parahippocampal place area (PPA; located within the parahippocampal cortex) and the hippocampus (Barense et al., 2010; Canário et al., 2016; Epstein et al., 1999; Graham et al., 2010; Lee, 2006; Lee et al., 2005, 2012; Mundy et al., 2013).

In aging, there is evidence for decreased neural integration and specialization of core face (e.g., in the FFA, PrC, ventral visual cortex) and place processing areas (e.g., in the hippocampus and parahippocampal cortex) (Berron et al., 2018; Dennis et al., 2008; Grady et al., 1995; Gutchess et al., 2005; Lee et al., 2011; Park et al., 2003, 2004, 2012; Payer et al., 2006; Zebrowitz et al., 2016) albeit compensatory activations are often reported in other brain regions, namely in frontal regions (Dennis et al., 2008; Grady et al., 1995; Gutchess et al., 2005; Lee et al., 2011; Park et al., 2003; Payer et al., 2006; Zebrowitz et al., 2016). Furthermore, impairments in processing of faces and places have been reported with age (Berron et al., 2018; Grady et al., 1995; Habak et al., 2008; Konar et al., 2013; Qian et al., 2017; Rousselet et al., 2009) and could reflect decrease neural specialization of the core object processing network (Berron et al., 2018; Dennis et al., 2008; Grady et al., 1995).

In the present study, we assessed age‐related effects on the microstructure of the fornix and the inferior longitudinal fasciculus (ILF) (Metzler‐Baddeley et al., 2011; Wakana et al., 2007) and on the macrostructure/volume of the hippocampus in relation to processing into working memory of visual object categories, that is, faces and places. More specifically, we sought to address the following questions (a) if age associates with micro‐ and macrostructural impairments in the fornix/ hippocampus and in the ILF and with decline in face and place processing and (b) test whether these age‐associated micro‐ and macrostructural impairments in hippocampal networks and in the ILF can relate to some decline in processing of places and faces, respectively. To test for specific contributions of the studied structures to face/place processing, we also studied a distinct tract (i.e., the anterior thalamic radiation [ATR]) and cognitive performance for other visual object categories (i.e., objects, bodies, and verbal material). The ATR contains fiber tracts connecting the prefrontal cortex with the thalamus, and while its fiber projections include the anterior thalamus, its putative role in visuospatial/scene processing abilities has been alternatively explained as part of an extended network connecting the anterior thalamus with the hippocampus and mammillary bodies (i.e., via the fornix) (Aggleton et al., 2010; Nelson et al., 2020; O'Mara and Aggleton, 2019).

**FIGURE 1:**
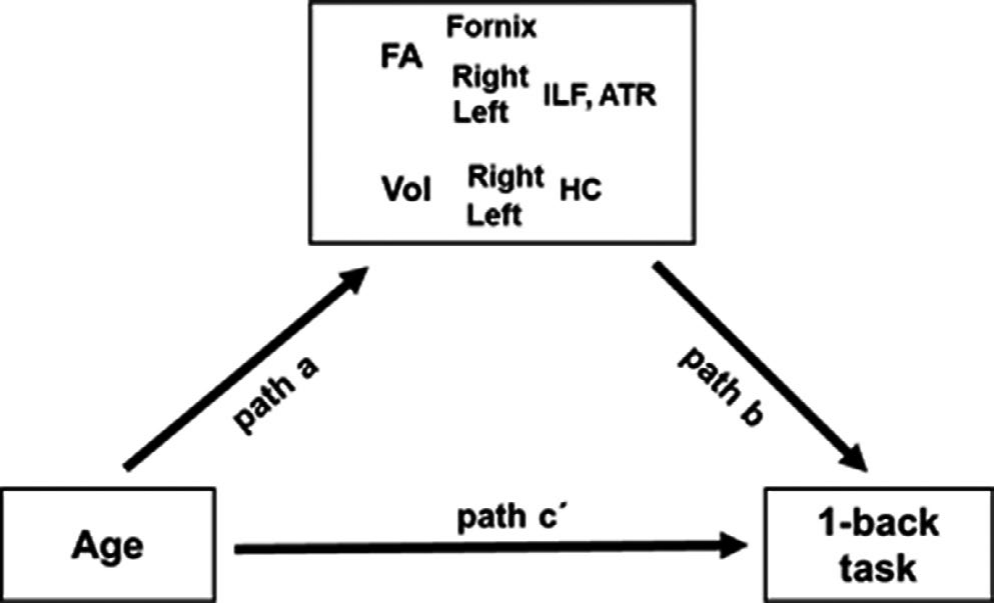
Schematic illustration of mediation model testing for indirect effects of microstructural (i.e., FA in the fornix and in the right and left ILF/ATR) and macrostructural (i.e., volume in the left and right hippocampus) variables on the direct effect of age on cognitive task performance (path c'). Path a measures the correlation between age and WM microstructure and GM macrostructure, and path b measures the partial correlation between WM microstructure, GM macrostructure, and cognitive performance (controlling out for age). The indirect effect is the product of a*b. HC, hippocampus; Vol, volume

To address the aforementioned questions, we first tested the potential effects of age on the micro‐ and macrostructure of the hippocampal, occipitotemporal connections and the ATR (as a control tract) and on cognitive performance as measured by 1‐back working memory tasks for visual object categories (faces, places, objects, bodies, and verbal material). We then tested in mediation models whether the inclusion of microstructural and macrostructural variables (i.e., fractional anisotropy (FA) in the fornix, FA in the left and right ILF/ATR and left/right hippocampal volume) mediated the direct effects of age on visual object category processing performance (see Figure 1). Thus, we sought to further highlight understanding of the aging of hippocampal and occipitotemporal connections and its implications in cognitive functioning, namely in face and place processing.

The fornix (i.e., the “extended hippocampal network”) represents the main hippocampal input/output projection and provides a route of communication between the hippocampus and sites such as the anterior thalamus, the mammillary bodies, and the middle prefrontal cortex (Aggleton, 2012; Aggleton et al., 2010). Fornix microstructural degradation constitutes an important biomarker in predicating conversion from healthy aging to early onset dementia and the associated impairments in episodic memory abilities that have been observed with age and in early Alzheimer disease (AD) (Douet & Chang, 2015; Metzler‐Baddeley et al., 2011; Mielke et al., 2012; Zhuang et al., 2013). Our interest in studying the fornix comes from studies showing that fornix transection in nonhuman primates impairs learning of object‐in‐place associations and conjunctions of spatial features both of which represent important facets in place processing and representation (Buckley et al., 2004; Gaffan, 1994; Wilson et al., 2008) and also from studies in young human adults reporting significant associations between fornix microstructure and place processing (Hodgetts et al., 2015; Postans et al., 2014). Furthermore, there is evidence for a right hippocampal involvement in visuospatial memory processing abilities (as opposed to the left hippocampus which is considered to be more involved in episodic and autobiographical memory) (Abrahams et al., 1997; Bohbot et al., 1998; Burgess et al., 2002; Ezzati et al., 2016).

In addition, we elected to study the ILF because of the anatomy of its occipitotemporal projections (i.e., it primarily connects extrastriate visual areas with regions in ventral anterior temporal cortex) and because its harbors and supports connectivity between the distinct nodes (e.g., FFA, PrC, OFA) implicated in perception of faces (Catani et al., 2003; Gschwind et al., 2012; Herbet et al., 2018; Pyles et al., 2013; Rivolta et al., 2013; Tusa & Ungerleider, 1985; Ungerleider & Haxby, 1994). For instance, Hodgetts et al. (2015) reported associations between ILF microstructure and (a) BOLD responses in face‐selective regions (i.e., in the FFA and PrC) and (b) performance in a face perception oddity task. Other important studies provided evidence for a link between impairments in right hemispheric ILF microstructure and prosopagnosia (i.e., deficits in face processing abilities despite normal vision and intelligence) (Grossi et al., 2014; Thomas et al., 2009; Tusa & Ungerleider, 1985).

We hypothesized that age would be associated with (a) decreases in FA in the fornix, while using the ATR as a negative control, and also in the ILF following previous observations of reduced neural specialization of face‐selective/occipitotemporal nodes with increasing age, (b) reduced volume in the hippocampus, and (iii) decline in visual object category processing (i.e., faces, places, and other object categories) (Berron et al., 2018; Cox et al., 2016; Dennis et al., 2008; Grady et al., 1995; Gutchess et al., 2005; Konar et al., 2013; Lee et al., 2011; Metzler‐Baddeley et al., 2011; Park et al., 2004, 2012). We also hypothesized that the fornix and the hippocampus (particularly in the right hemisphere) should have a mediating effect on the relationship between age and processing of place categories (Abrahams et al., 1997; Barense et al., 2010; Berron et al., 2018; Burgess et al., 2002; Graham et al., 2010; Hodgetts et al., 2015; Lee et al., 2012; Postans et al., 2014), whereas the ILF ought to have a mediating effect on the relationship between age and processing of faces (Grill‐Spector, 2003; Grill‐Spector et al., 2004, 2017; Grossi et al., 2014; Herbet et al., 2018; Hodgetts et al., 2015; Rivolta et al., 2013; Thomas et al., 2009).

## 2 METHODS

### 2.1 Participants

A total of 57 healthy volunteers (29 males and 28 females, aged 23–79 years) were recruited to take part in the study. Subjects had normal or corrected to normal vision and had no history of neurological and psychiatric disorders. All participants underwent assessment with the Montreal Cognitive Assessment test (MoCA) (Nasreddine et al., 2005) in order to screen for and exclude for cognitive impairment. Participants were excluded from the study when performance in the MoCA test was below more than 2 standard deviations from the respective mean in accordance to individual's age and education (Freitas et al., 2011). Working memory was assessed with 1‐back tasks (Owen et al., 2005). Two of the participants did not perform the 1‐back tasks. Thus, 55 cognitive and 57 MRI datasets were available for the final analyses. Table 1 provides a summary of background demographic information and cognitive test results for a total of 55 participants with complete MRI and cognitive datasets (i.e., excluding those 2 participants who did not perform the 1‐back tasks). All participants gave their written informed consent for the study, in accordance to the Declaration of Helsinki, and approval was obtained by the Ethics Committee of the Faculty of Medicine of the University of Coimbra.

**TABLE 1:** Participants’ demographics and cognitive profile

Demographics/cognition	
*N*	55
Age	*M* = 46.5, *SD* = 16.6 (range: 23–79 years)
Sex	27 male, 28 female
Years of education	*M* = 15, *SD* = 3.8 (range: 4–20 years)
General cognitive profile	
MoCA^a^	*M* = 27, *SD* = 2.2
1‐back tasks (stimulus)	
Faces	*M* = 80.9, *SD* = 17
Places	*M* = 83.3, *SD* = 17

Abbreviations: *M*, mean; *SD*, standard deviation.

^a^ One participant could not perform the MoCA test. Please note that the scores from the 1‐back tasks represent transformed *d* prime (*d*') scores of response accuracy.

### 2.2 Experimental task: Stimulus and procedures

**FIGURE 2:**
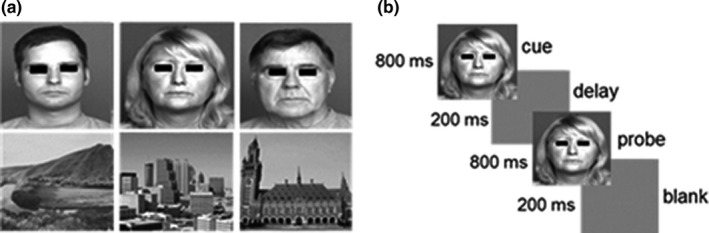
(a) Examples of faces and places subcategories of stimuli (b) Illustration of the 1‐back task as follow: cue (800 ms) + delay (200 ms) + probe (800 ms) + blank screen (200 ms). Participants were asked to press a button everytime cue and probe matched and to abstain from pressing any button whenever cue and probe did not match. Note that each individual stimulus could either serve as cue and/or probe

Visual processing was assessed for faces and places and other stimulus categories. The stimulus categories consisted of grayscale images. Each category contained 3 different subtypes of stimuli (see Figure 2a for examples). More specifically, faces categories were composed of young, middle, and old faces and were obtained from the FACES database (Ebner et al., 2010); places categories comprised landscapes, buildings, and skylines and were obtained from both online searches and a database of the computational visual cognition laboratory (Figure 2a) (Oliva & Torralba, 2001). Images were equalized in terms of luminance with the SHINE toolbox (Willenbockel et al., 2010). This procedure calculates a globally equalized luminance based on the average of the image input matrices. In order for the Shine procedure to be correct, a verified luminance‐corrected matrix needs to be calculated based on the SpectroColorimeter PR‐650 (Canário et al., 2016).

The experimental task was composed by two runs (versions A and B) with stimulus presented randomly in a block‐design paradigm. Individual runs were composed by 18 blocks with a duration of 9 min and 17 s. Individual blocks had a duration of 20 s and were composed of 20 images that always belonged to the same subcategory of stimuli (e.g., regarding the face categories a single block would always be composed of either just young faces, middle aged faces, or old faces). Each block was separated by a 10 s interval filled with a uniform greyscale image with no stimulus.

As noted above, other stimulus categories beyond faces and places were tested in the current experiment (i.e., objects, bodies, verbal material, and also scrambled versions of visual objects). In this study, these stimulus categories (with the exception of scrambled stimuli which represent meaningless abstract material) served as control to test for specific associations between hippocampal/ILF micro‐ and macrostructure and face and place processing (see Figure S1).

During the 1‐back tasks, individual images were presented for 800 ms with a 200 ms gap and participants were asked to press a button with their dominant hand every time the image being presented was the same that had been presented before (Figure 2b). The order of blocks was presented in a pseudo‐randomized fashion. There were four repetitions of images within each block. Before starting the actual experiment, subjects performed a brief training session in order to guarantee that they understood the task demands and to be familiarized with the stimuli.

Visual stimuli were presented using Presentation 17.1 software (Neurobehavioral systems) on a uniformly black background. Stimuli were presented using a Fujitsu PC (1,920 × 1,080) onto an LCD screen. The images size used to build the stimuli was 544 × 544 pixels and subtended approximately 11° × 11° of visual field.

### 2.3 MRI data acquisition

MRI data were acquired in a 3 Tesla Siemens Magnetom TrioTim scanner at the Institute of Nuclear Sciences Applied to Health (ICNAS) using a 12‐channel birdcage head coil. The session started with one 3D anatomical MPRAGE (rapid gradient‐echo) sequence T1‐weighted with a 1.0 × 1.0 × 1.0 mm voxel resolution, repetition time (TR) 2,530 ms, echo time (TE) 3.42 ms, and field of view (FOV) 256 × 256 mm. The MPRAGE sequence comprised 176 slices, a flip angle of 7°, and an inversion time of 1,100 ms. The protocol also included a diffusion tensor imaging (DTI) sequence with the following parameters: TR/TE = 7,800/90 ms, number of excitations = 1, matrix = 96 × 96 × 63 contiguous axial slices, isotropic voxel resolution = 2 × 2 × 2 mm^3^, bandwidth = 1,628 Hz/pixel, echo spacing = 0.72 ms, 63 noncollinear directions, one scan without diffusion weighting (*b* = 0 s/mm^2^, *b*0), and *b*‐value = 1,000 s/mm^2^).

### 2.4 Diffusion MRI processing and tractography

The diffusion‐weighted MRI images were corrected for subject motion, eddy‐current‐induced distortions, and EPI deformations by registering each image volume to the high‐resolution T1‐weighted anatomical images (Irfanoglu et al., 2012; Soares et al., 2013) with appropriate reorientation of the encoding vectors (Leemans & Jones, 2009) in ExploreDTI (version 4.8.6; Leemans et al., 2009).

**FIGURE 3:**
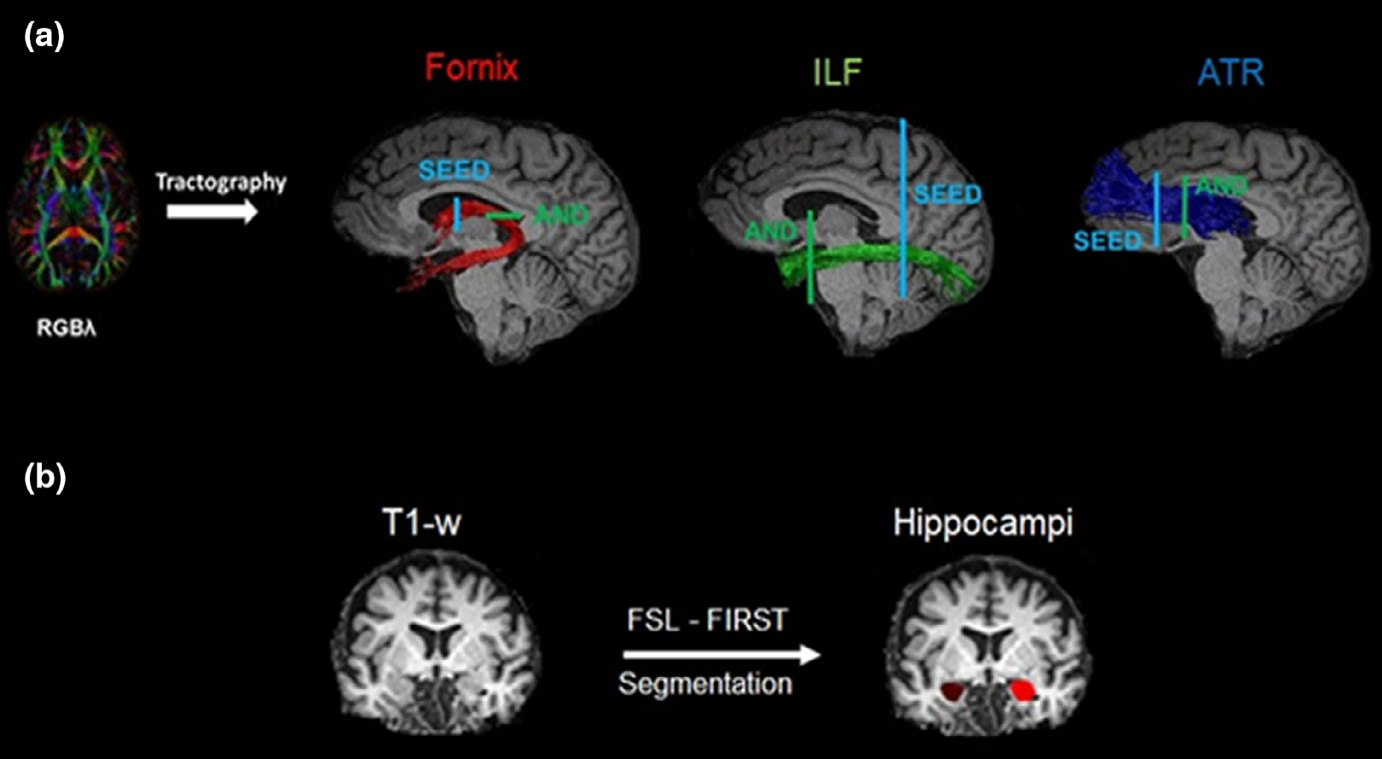
(a) Displays sagittal views of the reconstructed WM pathways of interest on a T1‐weighted image of one participant. The fornix, inferior longitudinal fasciculus (ILF), and the anterior thalamic radiation (ATR) were reconstructed with constrained spherical deconvolution (CSD) based deterministic tractography on color coded principal direction maps (RGBλ). Placement of SEED gates is shown in blue, and AND gates are shown in green. (b) Gray matter (GM) left and right hippocampal volumes were segmented from T1‐weighted images (T1‐w) with FSL‐FIRST toolbox

The fornix, ILF, and ATR were isolated using CSD‐based deterministic tractography (Jeurissen et al., 2011) with the following parameters: uniform whole‐brain seeding with 2 mm resolution, step size 1 mm, fiber orientation distribution threshold of 0.1, and maximum angle deviation of 30°. Three‐dimensional fiber reconstructions of the WM tracts of interest were made by applying waypoint ROI gates (“AND”, “SEED” and “NOT” gates following Boolean logic) to isolate specific tracts from the whole‐brain CSD‐based tractography data. Displays of the fiber tracts and placement of SEED and AND ROIs are shown in Figure 3a.

The reconstruction of the fornix followed the protocol by Metzler‐Baddeley et al. (2011). A SEED ROI was placed on the coronal slice around the area where the anterior pillars enter into the main body of the fornix. An AND ROI was placed on the axial plane encompassing the crus of the fornix in both hemispheres around the inferior part of the splenium of the corpus callosum. NOT gates were placed around the protruding areas that are not part of the fornix shape.

The reconstruction of the ILF and the ATR was based on the anatomical atlas by Wakana et al.^2007^. To segment the ILF, first the posterior end of the cingulum was identified and from that position after reverting into the coronal plane a SEED gate was placed around the entire hemisphere. The AND ROI was drawn on a coronal plane around its most posterior section where the temporal lobe is no longer connected to the frontal lobe. Here, the AND ROI was drawn around the entire temporal lobe. Like in the fornix, NOT gates were drawn on projections that are not part of the ILF.

For segmentation of the ATR, first the middle section of the corpus callosum was identified, and from that position after reverting to the coronal plane, a SEED gate was placed around the anterior limb of the internal capsule. In the same coronal plane, an AND gate was drawn around the entire thalamus at the level of the anterior edge of the pons. NOT gates were drawn to eliminate projections outside the ATR. In both cases (ILF and ATR), the protocol of segmentation was applied to both hemispheres.

Following the reconstruction of the tracts of interest (Figure 3a), average indices of FA were extracted (for the fornix and for the left and right ILF/ATR).

### 2.5 Extraction of hippocampal volumes from T1‐weighted anatomical images

GM subcortical volumes of the left and right hippocampus were extracted from individual T1‐weighted images using the FMRIB Software Library (FSL) FIRST registration and segmentation tool (Patenaude et al., 2011; www.fsl.fmrib.ox.ac.uk/fsl/fslwiki/FIRST; Figure 3b). The FIRST procedure involves as a first step the registration of each individual's T1‐weighted image to the Montreal Neurological Institute (MNI) standard template with affine registration. During this step, voxels outside subcortical regions are excluded using an MNI subcortical mask. FIRST then applies a Bayesian model of shape recognition to perform segmentation of subcortical structures. The segmented images were uploaded onto the original T1‐weighted images and were visually inspected for correct registration for all participants. Quantitative volume measures from the hippocampal segmentations were extracted using the FSL statistics tool.

In addition, all FIRST subcortical volumes were corrected for head size with the volumetric scaling factor derived from SIENAX version 2.6 (part of FSL 5.0, http://www.fmrib.ox.ac.uk/fsl; Smith et al., 2001). This involved skull extraction with BET and an affine registration to the Montreal Neurological Institute (MNI) standard template. The volumetric scaling factor/value was extracted and used as the normalization factor to obtain head size corrected volumes (i.e., by multiplying the volumetric scaling value with the subcortical volumes obtained from FIRST).

### 2.6 Statistical analyses

All statistical analyses were carried out in IBM SPSS statistical package (version 24) and the PROCESS computational tool for mediation analysis (Hayes, 2012). All data were inspected for outliers defined as values larger or smaller than three times the standard deviation from the mean of the microstructural, volumetric, and cognitive data. The data were also checked for normality with Shapiro–Wilk tests.

First, to acquire more sensitive measures of response accuracy from the 1‐back tasks, the original raw scores of response accuracy were transformed in order to obtain *d* prime (*d*') scores of accuracy (Wickens, 2010). For each individual subject, the hit rate (*H*) and the false alarms rates (FAR) were calculated and then transformed to *z*‐scores. Subsequently, *d* prime (*d')* scores were obtained using the following formula: *d*' = *z* (*H*) – *z* (FAR). The *d* prime (*d´*) scores of response accuracy were used as outcome measures of performance for the 1‐back control tasks in all of the analyses.

Omnibus multivariate regression analysis was conducted to test effects of age, sex, and years of education simultaneously on (a) all MRI measures (computed as follow: FA fornix + FA right ILF + FA left ILF + FA right ATR + FA left ATR + volume right hippocampus + volume left hippocampus/7) and (b) all cognitive measures (computed as: *d* prime scores faces + *d* prime scores places + *d* prime scores objects + *d* prime scores bodies + *d* prime scores verbal/5).

Effects of age on (a) WM microstructure and (b) GM macrostructure were tested with separate Pearson's correlations. Relationships between age and cognition were tested with Spearman's rho correlations. In addition, separate Pearson's correlations analysis was conducted to test (a) relationships between right hippocampal volume and WM microstructure and (b) relationships between left hippocampal volume and WM microstructure. The correlation analysis was corrected for multiple comparisons with the Bonferroni correction with a family‐wise alpha level of 5% (two‐tailed) leading to a corrected *p*‐value of ≤.01 for five correlations between age and WM microstructure, *p* ≤.025 for two correlations between age and GM macrostructure, *p* ≤ .01 for five correlations between age and cognitive performance, and *p* ≤ .01 for five correlations between right and/or left hippocampal volume and WM microstructure.

Subsequently, linear mediation analysis was conducted to test for the indirect effects a*b of WM microstructural and GM subcortical mediator variables (i.e., FA in the fornix, FA in the left and right ILF/ATR and volume in the left and right hippocampus) on the direct effects c’ of age on cognitive performance (see Figure 1 for illustration of the model). The significance of indirect and direct effects was assessed with a 95% confidence interval based on bootstrapping with 5,000 replacements.

## 3 RESULTS

Overall, participants exhibited a score of 27 (out of a total of 30) in the MoCA test, and cognitive performance for faces and place categories was of 80.9 and 83.3, respectively (out of a total of 100; see Table 1, for other details). The microstructural and volumetric data conformed to normality for which Pearson's correlations were conducted, while the cognitive data did not conform to normality for which nonparametric Spearman´s rho correlations were conducted. One participant (aged 68) left hippocampal volume was identified as an outlier. Results will be reported after and before outlier exclusion.

### 3.1 Omnibus multivariate regression

Multivariate regression analysis testing simultaneously for omnibus effects of age, sex, and years of education as independent variables on all MRI outcome measures as dependent variables showed that age, sex, and years of education together explained a significant amount of variance in overall micro‐ and macrostructure (*F* (3,53) = 6.4, *p* = .001, *R^2^* = 0.265). At the individual predictor level, there were effects of age (*t* (56) = −3.4, *p* = .001) and sex (*t* (56) = −2.9, *p* = .006) showing that micro‐ and macrostructural integrity was higher for females in comparison with males. There were no effects of years of education (*t* (56) = −1.8, *p* = .074) on overall MRI measures.

In addition, age, sex, and years of education simultaneously also explained a significant amount of variance in overall cognitive performance (*F* (3, 51) = 3.6, *p* = .02, *R^2^* = 0.18). At the individual predictor level, only an effect of age was observed (*t* (54) = −2.8, *p* = .007). There were no effects of sex (*t* (54) = −0.56, *p* = .58) and years of education (*t* (54) = 0.34, *p* = .73) on overall cognitive performance.

### 3.2 Effects of age on WM microstructure, GM macrostructure, and cognition

**FIGURE 4:**
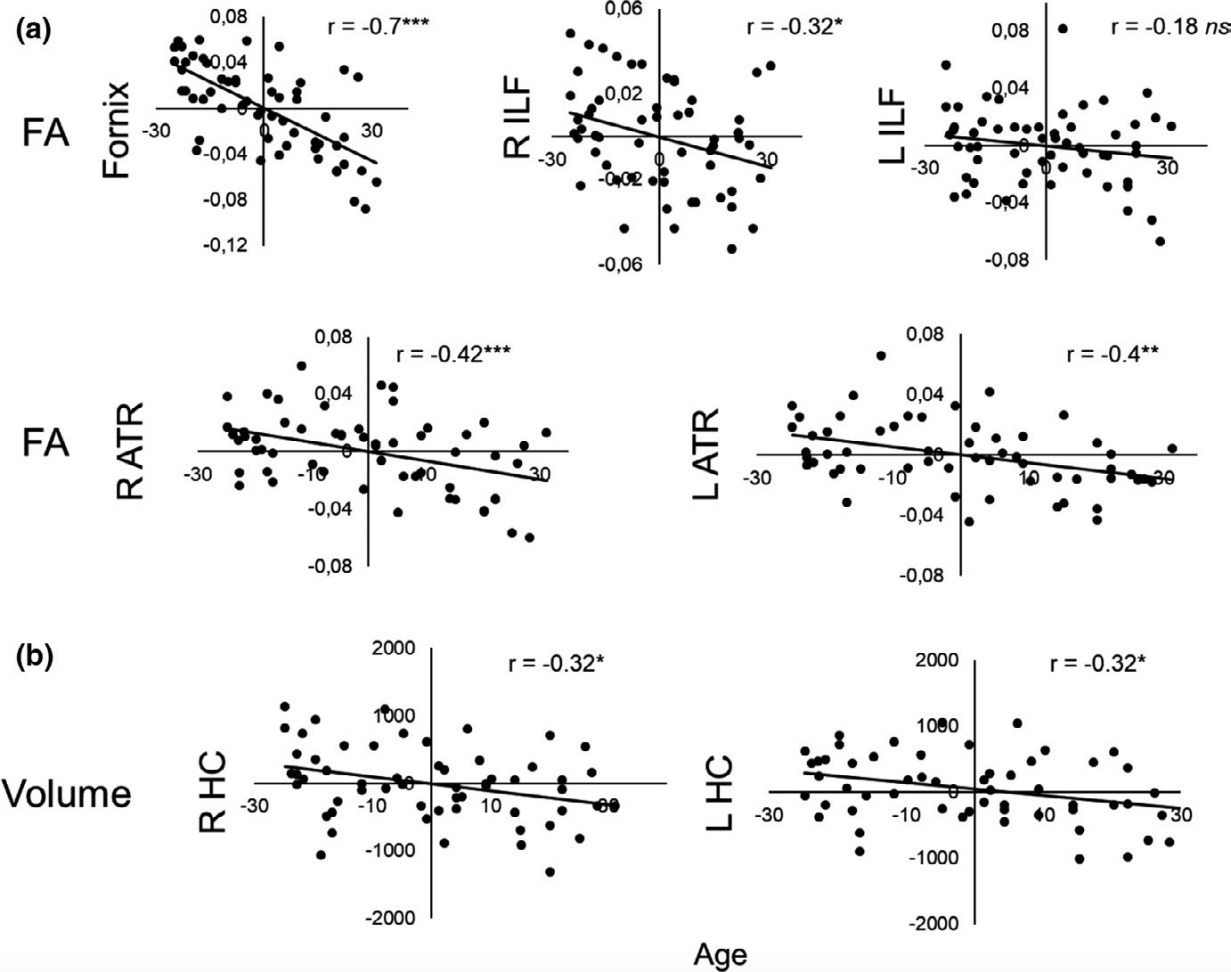
Scatterplots displaying the negative Pearson correlations between age and (a) WM microstructure and (b) GM macrostructure (controlling out for sex)(*n* = 57). Plotted age and WM microstructure and GM macrostructure measures represent unstandardized residuals scores (i.e., regressed against sex). Please note the correlation plots between age and left hippocampal volume are based on *n* = 56 subjects (i.e., after outlier exclusion). Abbreviations: R ILF, right inferior longitudinal fasciculus; L ILF, left inferior longitudinal fasciculus; R ATR, right anterior thalamic radiation; L ATR, left anterior thalamic radiation; R HC, right hippocampus; L HC, left hippocampus; ns, nonsignificant. *p≤.05; **p≤.01, ***p ≤.001

There were statistically significant negative correlations between age and FA in the fornix (*r* = −0.7, *p* < .001, Bonferroni‐corrected *p* ≤ .01), right ATR (*r* = −0.42, *p* = .001, Bonferroni‐corrected *p* ≤ .01), and in the left ATR (*r* = −0.4, *p* =.003, Bonferroni‐corrected *p* ≤ .01). There was also a trend (i.e., significant at uncorrected level) toward a negative association between age and FA in the right ILF (*r* = −0.32, *p* = .018). No significant associations were observed between age and FA in the left ILF (*r* = −0.18, *p* = .2) (Figure 4a).

There was a statistically significant negative relationship between age and volume in the right (*r* = −0.32, *p* = .018, Bonferroni‐corrected *p* ≤ .025) and left hippocampus (*r* = −0.32, *p* = .017, Bonferroni‐corrected *p* ≤ .025, *n* = 56) (Figure 4b). The relationship between age and left hippocampal volume held significant when the outlier was included in the analysis (*r* = −0.35, *p* = .008, Bonferroni‐corrected *p* ≤ .025).

In addition, there was a statistically significant negative relationship between age and cognitive performance for face categories and a trend toward a negative association between age and cognitive performance for places categories (Table 2; Figure S2). Lastly, there was also a statistically significant negative association between age and cognitive performance for object categories (*r* = −0.45, *p* = .001, Bonferroni‐corrected *p* ≤ .01) and trends toward negative associations between age and cognitive performance for bodies (*r* = −0.31, *p* = .02) and verbal categories (*r* = −0.32, *p* = .016).

**TABLE 2:** Spearman's correlation coefficients between age and cognitive performance for faces and places categories (*n* = 55)

1‐back tasks (stimulus)	Age
Faces	*r* = −0.36, *p* =.006
Places	*r* = −0.32, *p* =.016

### 3.3 Relationships between microstructural and macrostructural measures

Subsequently, Pearson's correlation analysis tested the relationships between macrostructural (i.e., volume in left and right hippocampus) and microstructural (i.e., FA in the fornix and in the left and right ILF/ATR) MRI measures.

**FIGURE 5:**
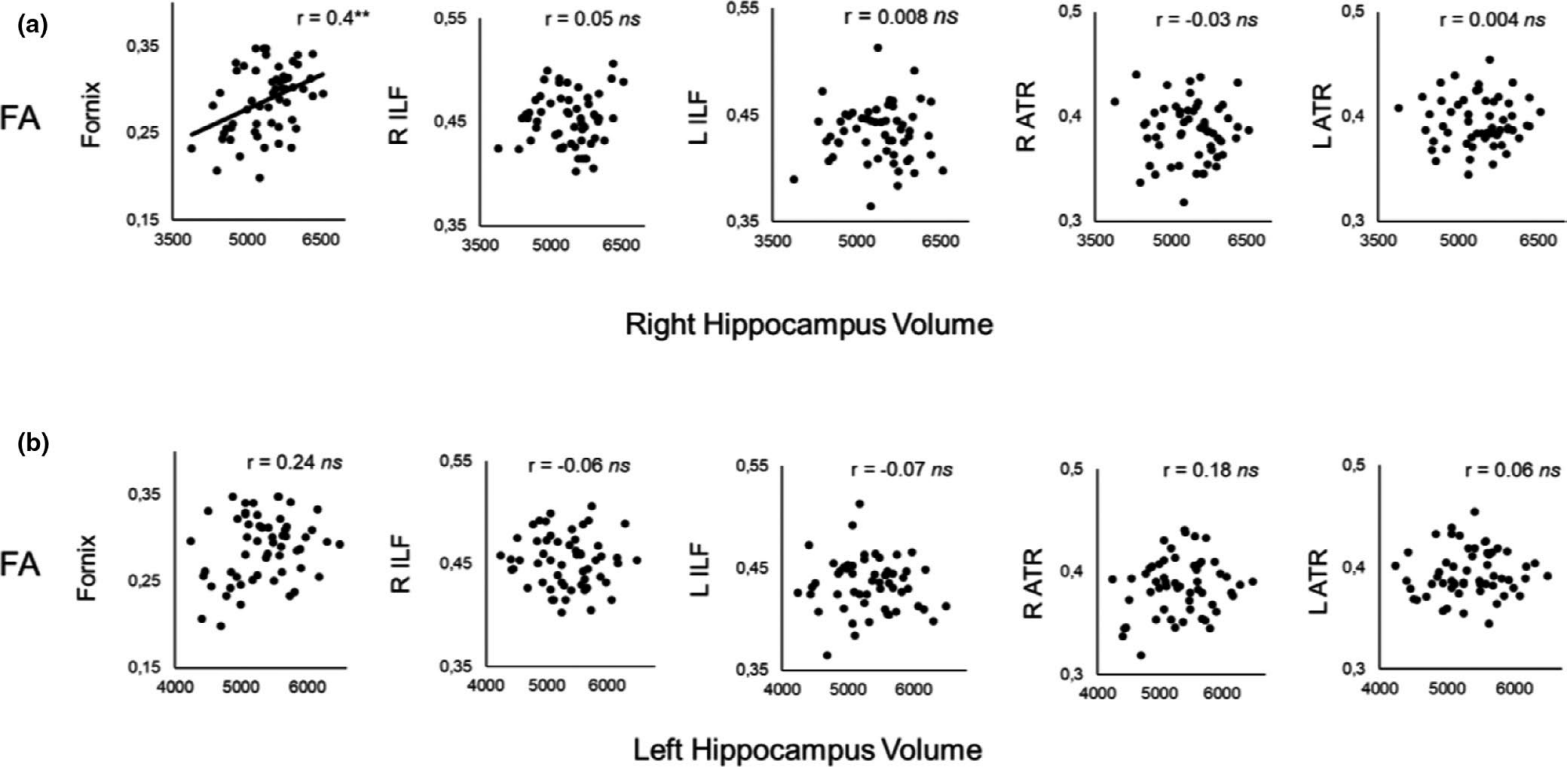
Scatterplots depicting the Pearson correlations between (a) right hippocampal volume and WM microstructure (*n* = 57) and (b) left hippocampal volume and WM microstructure (*n* = 56)

There was a statistically significant positive association between right hippocampal volume and fornix FA (*r* = 0.4, *p* = .002, Bonferroni‐corrected *p* ≤ .01). There were no significant associations between right hippocampal volume and FA in the right ILF (*r* = 0.05, *p* = .7), left ILF (*r* = 0.008, *p* = .95), right ATR (*r* = −0.03, *p* = .81), and in the left ATR (*r* = 0.004, *p* = .98) (Figure 5a).

In addition, there were no significant associations between left hippocampal volume and FA in the fornix (*r* = 0.24, *p* = .07), right ILF (*r* = −0.06, *p* = .7), left ILF (*r* = −0.07, *p* = .63), right ATR (*r* = 0.18, *p* = .19), and in the left ATR (*r* = 0.06, *p* = .67) (Figure 5b).

Please note that the relationship between left hippocampal volume and fornix FA turned significant (i.e., at uncorrected level) after inclusion of the outlier in the analysis (*r* = 0.31, *p* = .02). No other significant associations were observed.

### 3.4 Mediation analysis testing the contribution of hippocampal, occipitotemporal, and fronto‐thalamic micro‐ and macrostructure on age–cognitive relationships

**FIGURE 6:**
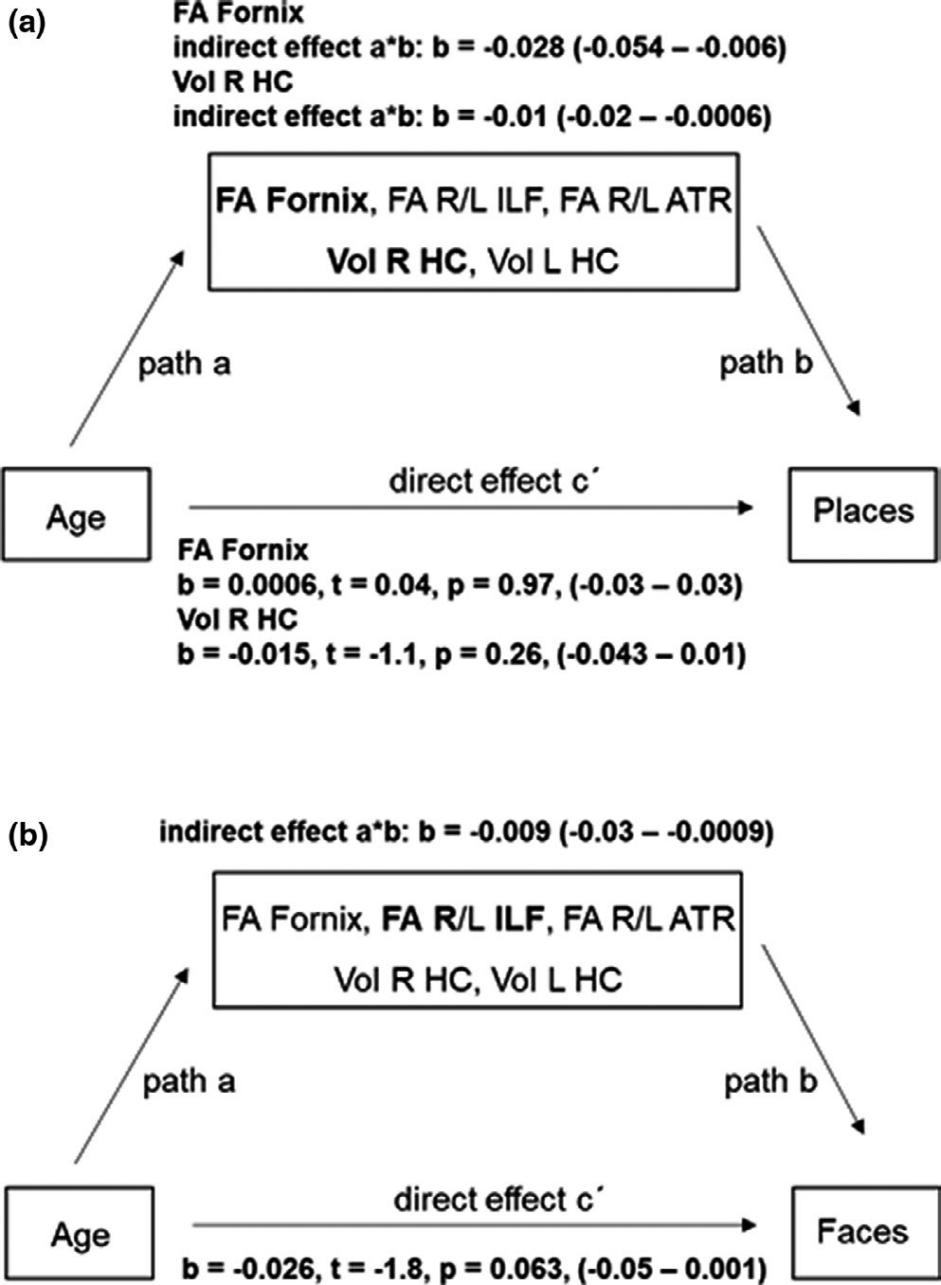
Schematic illustration and outcome of the mediation analysis (*n* = 55). (a) Fornix and right hippocampal mediators fully mediated the direct effects of age on place category performance (highlighted in bold). (b) Right ILF mediator variable fully mediated the direct effects of age on face category performance (highlighted in bold). 95% confidence interval in brackets was based on bootstrapping with 5,000 replacements. *b*, unstandardized coefficients, Vol, volume

Mediation analysis testing for indirect effects of hippocampal, occipitotemporal, and fronto‐thalamic micro‐ and macrostructural mediator variables on the direct effects of age on cognitive performance showed that the fornix and right hippocampal mediator variables fully mediated the relationship between age and processing of place categories (see Figure 6a, highlighted in bold). In addition, right ILF microstructure fully mediated the relationship between age and processing of face categories (Figure 6b, highlighted in bold).

There were no other mediator effects of micro‐ and macrostructural variables on age–cognition relationships (see Table S1).

## 4 DISCUSSION

In the present study, we studied age‐related effects on the microstructure of the fornix and the inferior longitudinal fasciculus (ILF) and on the macrostructure/volume of the hippocampus in relation to cognitive processing of visual object categories, that is, faces and places. In particular, we sought to determine (a) whether age associates with micro‐ and macrostructural impairments in the fornix/hippocampus and in the ILF and with decline in face and place processing and (b) test whether these age‐associated micro‐ and macrostructural impairments in hippocampal networks and in the ILF can relate to some decline in processing of places and faces, respectively. To test for specific of contributions of the studied structures to face/place processing, we also studied a distinct tract (the ATR) and cognitive performance for other visual object categories (i.e., objects, bodies, and verbal material).

To address the proposed questions, we first tested the potential effects of age on the micro‐ and macrostructure of the hippocampal, occipitotemporal, and fronto‐thalamic connections and on cognitive performance as measured by 1‐back tasks for visual object categories (faces, places, objects, bodies, and verbal material). We then tested in mediation models whether the inclusion of microstructural and macrostructural variables (i.e., fractional anisotropy (FA) in the fornix, FA in the left and right ILF/ATR and left/right hippocampal volume) mediated the direct effects of age on visual object category processing performance.

We found that age associates with (a) decreases in FA in the fornix, in the right ILF (but not in the left ILF), and in the right and left ATR (b) reduced volume in the right and left hippocampus and (c) decline in visual object category processing. Most notably, mediation analysis suggests that age‐associated micro‐ and macrostructural impairments in the fornix/right hippocampus and in the right ILF, respectively, relate to some decline in place and face category processing. There were no adding contributions of hippocampal and ILF structures to processing of other visual object categories nor the ATR (the control tract) exhibited any associations with face/place processing or any other visual object categories. Together, these findings support specific contributions of the fornix and the right hippocampus in visuospatial scene processing abilities and of the long‐range right hemispheric occipitotemporal network in face category processing.

Importantly, structural parameters of the fornix and the right hippocampus were strongly correlated with each other. It is likely that in older age, micro‐ and macrostructural impairments within hippocampal structures can lead to some deficit in visuospatial processing abilities which are necessary attributes for place processing and have been shown to depend on the integrity of the fornix (i.e., in the animal and in humans) (Buckley et al., 2004; Dumont et al., 2015; Gaffan, 1994; Hofstetter et al., 2013; Wilson et al., 2008) and on the integrity of the right hippocampus (Abrahams et al., 1997; Bohbot et al., 1998; Burgess et al., 2002; Ezzati et al., 2016; O’Keefe et al., 1998). The findings reported here further support a role of the right hippocampus in perception and visuospatial processing abilities that is contrary to that of the left hippocampus (Abrahams et al., 1997; Bohbot et al., 1998; Burgess et al., 2002; Ezzati et al., 2016). Thus, some level of deficit in the recognition of place categories in the older may also reflect the decreases in neural activation/specialization within the hippocampus to place stimuli (Dennis et al., 2008; Gutchess et al., 2005; Park et al., 2003). In addition to evidence provided by functional activation patterns, functional connectivity data also provide further support for aging effects. For instance, previous studies suggested decreases in neural recruitment and connectivity between hippocampal–prefrontal regions (a network supported by the fornix) to relate to deficits in the recognition of visual objects (e.g., places and faces) in older age (Dennis et al., 2008; Grady et al., 1995). More recently, Jiang et al. (2017) reported a direct positive association between neural selectivity within the hippocampal formation and performance in an episodic memory task in a group of healthy older adults. As a result, the authors argued that age‐associated decreases in neural selectivity might better reflect decline in cognitive function rather than compensation (Jiang et al., 2017, see also Park et al., 2010). Other important recent studies support the hypothesis that aging leads to decreased neural selectivity within place selective regions (e.g., in the PPA a region analogous to the hippocampus) and that such decreases in neural selectivity impact on cognitive performance (e.g., on reduced memory recognition for scenes), though these latter effects were found to be independent of age (Koen et al., 2019; Koen & Rugg, 2019; Srokova et al., 2020).

In addition, some degree of impairment in face recognition in the older may reflect the decreased levels of neural selectivity and specialization of face‐selective nodes (e.g., in the FFA, PrC, OFA) (Berron et al., 2018; Koen & Rugg, 2019; Lee et al., 2011; Park et al., 2004, 2012; Zebrowitz et al., 2016) and/or reduced connectivity among these nodes that are supported by the ILF (Gschwind et al., 2012; Pyles et al., 2013). For instance, a highly relevant study by Rieck et al. (2020) linking structural with functional connectivity found age‐related reductions in ILF anisotropy to predict decreases in neural selectivity to face stimuli in a fusiform region and across the extended ventral visual cortex. Furthermore, as noted earlier, Hodgetts et al. (2015) found that ILF microstructure associated not only with performance in a face perception oddity task but also with BOLD responses in face‐selective regions (i.e., in the FFA and PrC) suggesting that the anatomical links supported by the ILF can be of critical importance to face processing (Hodgetts et al., 2015; Scherf et al., 2014).

Secondly, the findings reported here suggest that aging of a right hemispheric ILF microstructure contributes to some deficit in face processing following previous reports of significant associations between impairments in right hemispheric ILF and prosopagnosia (Grossi et al., 2014; Thomas et al., 2009). Thus, our findings are also consistent with reports of structural and functional coupling between face‐responsive regions (e.g., PrC – FFA, FFA – OFA) in the right hemisphere (Gschwind et al., 2012; O’Neil et al., 2013, 2014) and in response to face stimuli (O’Neil et al., 2013, 2014) and with large functional neuroimaging evidence supporting preferential responses of a subset of right hemispheric occipitotemporal nodes (e.g., FFA, OFA) to face categories in comparison with other visual objects (e.g., Canário et al., 2016; Grill‐Spector et al., 2017; Pitcher et al., 2011).

Previous studies have debated about the limitations of drawing strong conclusions from a mediation analysis based on a cross‐sectional study design as opposed to a longitudinal one (Lindenberger et al., 2011). Nonetheless, despite these limitations, note that in context of the present study we report specific contributions/mediator effects of the hippocampal and ILF structures (but not of the ATR the control tract) to processing of places and faces categories as opposed to other visual object categories.

The specificity of contributions of occipitotemporal and hippocampal networks to face and place processing, respectively, supports category‐specific mechanisms of processing beyond nonspecific attentional effects, because category related performance was task matched. Thus, faces are likely to rely mainly on some sort of holistic/automated mechanism of processing driven by low‐level occipitotemporal networks while places likely rely on visuospatial mechanisms driven by hippocampal systems (Canário et al., 2016; Hodgetts et al., 2015; Postans et al., 2014; Schiltz & Rossion, 2006).

Because FA is affected by multiple proprieties of tissue microstructure, including axonal density/diameter and myelination, it is not possible in the context of the present study to attribute age‐associated decline in cognition to a particular propriety of tissue microstructure (Alexander et al., 2007; Jones et al., 2013). Thus, an interesting avenue for future studies would be to expand the use of imaging modalities beyond DTI (e.g., the composite hindered and restricted model of diffusion [CHARMED], magnetization transfer [MT], and myelin water imaging [MWI]) to better characterize the nature of microstructural impairments associated with cognitive decline in aging (Assaf & Basser, 2005; Heath et al., 2018).

## 5 CONCLUSION

The findings from the present study suggest that age‐related micro‐ and macrostructural impairments in the fornix and right hippocampus, respectively, associate with some decline in place processing in keeping with important roles of the extended hippocampal system and the right hippocampus in visuospatial processing abilities and scene perception (Buckley et al., 2004; Burgess et al., 2002; Dumont et al., 2015; Ezzati et al., 2016; Gaffan, 1994; Hodgetts et al., 2015; Hofstetter et al., 2013; Wilson et al., 2008). Conversely, age‐related microstructural impairments in right hemispheric ILF seem to contribute to some deficit in face category processing expanding on previous studies on prosopagnosia patients (Grossi et al., 2014; Thomas et al., 2009) and on substantial functional neuroimaging reports of enhanced activity and coupling of right hemispherical occipitotemporal nodes to face categories (Grill‐Spector et al., 2017; O’Neil et al., 2013, 2014; Pitcher et al., 2011).

## CONFLICT OF INTEREST

None declared.

## AUTHOR CONTRIBUTION

MCB and JBT involved in conceptualization and writing–reviewing and editing. MCB involved in data curation and supervision. JBT involved in writing–original draft preparation and visualization. MCB, NC, LJ, and JBT involved in investigation.

### PEER REVIEW

The peer review history for this article is available at https://publons.com/publon/10.1002/brb3.2127.

## Supporting information

Fig S1

Fig S2

Table S1

## Data Availability

Data are available upon request to the corresponding author or the Ethics Institutional Board.

## References

[brb32127-bib-0001] Abrahams, S., Pickering, A., Polkey, C. E., & Morris, R. G. (1997). Spatial memory deficits in patients with unilateral damage to the right hippocampal formation. Neuropsychologia, 35, 11–24. 10.1016/S0028-3932(96)00051-6 8981373

[brb32127-bib-0002] Aggleton, J. P. (2012). Multiple anatomical systems embedded within the primate medial temporal lobe: Implications for hippocampal function. Neuroscience and Biobehavioral Reviews, 36, 1579–1596. 10.1016/j.neubiorev.2011.09.005 21964564

[brb32127-bib-0003] Aggleton, J. P., O’Mara, S. M., Vann, S. D., Wright, N. F., Tsanov, M., & Erichsen, J. T. (2010). Hippocampal‐anterior thalamic pathways for memory: Uncovering a network of direct and indirect actions. European Journal of Neuroscience, 31, 2292–2307. 10.1111/j.1460-9568.2010.07251.x PMC2936113 20550571

[brb32127-bib-0004] Alexander, A. L., Lee, J. E., Lazar, M., & Field, A. S. (2007). Diffusion tensor imaging of the brain. Neurotherapeutics: the Journal of the American Society for Experimental NeuroTherapeutics, 4, 316–329. 10.1016/j.nurt.2007.05.011 17599699 PMC2041910

[brb32127-bib-0005] Assaf, Y., & Basser, P. J. (2005). Composite hindered and restricted model of diffusion (CHARMED) MR imaging of the human brain. NeuroImage, 27, 48–58. 10.1016/j.neuroimage.2005.03.042 15979342

[brb32127-bib-0006] Barense, M. D., Henson, R. N. A., Lee, A. C. H., & Graham, K. S. (2010). Medial temporal lobe activity during complex discrimination of faces, objects, and scenes: Effects of viewpoint. Hippocampus, 20, 389–401. 10.1002/hipo.20641 19499575 PMC2912509

[brb32127-bib-0007] Behrmann, M., & Plaut, D. C. (2013). Distributed circuits, not circumscribed centers, mediate visual recognition. Trends in Cognitive Sciences, 17, 210–219. 10.1016/j.tics.2013.03.007 23608364

[brb32127-bib-0008] Berron, D., Neumann, K., Maass, A., Schütze, H., Fliessbach, K., Kiven, V., Jessen, F., Sauvage, M., Kumaran, D., & Düzel, E. (2018). Age‐related functional changes in domain‐specific medial temporal lobe pathways. Neurobiology of Aging, 65, 86–97. 10.1016/j.neurobiolaging.2017.12.030 29454154

[brb32127-bib-0009] Bohbot, V. D., Kalina, M., Stepankova, K., Spackova, N., Petrides, M., & Nadel, L. (1998). Spatial memory deficits in patients with lesions to the right hippocampus and to the right parahippocampal cortex. Neuropsychologia, 6, 1217–1238. 10.1016/S0028-3932(97)00161-9 9842767

[brb32127-bib-0010] Buckley, M. J., Charles, D. P., Browning, P. G. F., & Gaffan, D. (2004). Learning and retrieval of concurrently presented spatial discrimination tasks: Role of the fornix. Behavioral Neuroscience, 118, 138–149. 10.1037/0735-7044.118.1.138 14979790

[brb32127-bib-0011] Burgess, N., Maguire, E. A., & O’Keefe, J. (2002). The human hippocampus and spatial and episodic memory. Neuron, 35, 625–641. 10.1016/S0896-6273(02)00830-9 12194864

[brb32127-bib-0012] Canário, N., Jorge, L., Loureiro Silva, M. F., Alberto Soares, M., & Castelo‐Branco, M. (2016). Distinct preference for spatial frequency content in ventral stream regions underlying the recognition of scenes, faces, bodies and other objects. Neuropsychologia, 87, 110–119. 10.1016/j.neuropsychologia.2016.05.010 27180002

[brb32127-bib-0013] Catani, M., Jones, D. K., Donato, R., & Ffytche, D. H. (2003). Occipito‐temporal connections in the human brain. Brain, 126, 2093–2107. 10.1093/brain/awg203 12821517

[brb32127-bib-0014] Cox, S. R., Ritchie, S. J., Tucker‐Drob, E. M., Liewald, D. C., Hagenaars, S. P., Davies, G., Wardlaw, J. M., Gale, C. R., Bastin, M. E., & Deary, I. J. (2016). Ageing and brain white matter structure in 3,513 UK Biobank participants. Nature Communications, 7, 13629. 10.1038/ncomms13629 PMC5172385 27976682

[brb32127-bib-0015] Dennis, N. A., Hayes, S. M., Prince, S. E., Madden, D. J., Huettel, S. A., & Cabeza, R. (2008). Effects of aging on the neural correlates of successful item and source memory encoding. Journal of Experimental Psychology: Learning Memory and Cognition, 34, 791–808. 10.1037/0278-7393.34.4.791 PMC2752883 18605869

[brb32127-bib-0016] Direito, B., Lima, J., Simões, M., Sayal, A., Sousa, T., Luehrs, M., Ferreira, C., & Castelo‐Branco, M. (2019). Targeting dynamic facial processing mechanisms in superior temporal sulcus using a novel fMRI neurofeedback target. Neuroscience, 406, 97–108. 10.1016/j.neuroscience.2019.02.024 30825583

[brb32127-bib-0017] Douet, V., & Chang, L. (2015). Fornix as an imaging marker for episodic memory deficits in healthy aging and in various neurological disorders. Frontiers in Aging Neuroscience, 6, 343. 10.3389/fnagi.2014.00343 25642186 PMC4294158

[brb32127-bib-0018] Dumont, J. R., Amin, E., Wright, N. F., Dillingham, C. M., & Aggleton, J. P. (2015). The impact of fornix lesions in rats on spatial learning tasks sensitive to anterior thalamic and hippocampal damage. Behavioural Brain Research, 278, 360–374. 10.1016/j.bbr.2014.10.016 25453745 PMC4274319

[brb32127-bib-0019] Ebner, N. C., Riediger, M., & Lindenberger, U. (2010). FACES‐a database of facial expressions in young, middle‐aged, and older women and men: Development and validation. Behavior Research Methods, 42, 351–362. 10.3758/BRM.42.1.351 20160315

[brb32127-bib-0020] Epstein, R., Harris, A., Stanley, D., & Kanwisher, N. (1999). The parahippocampal place area: Recognition, navigation, or encoding? Neuron, 23, 115–125. 10.1016/S0896-6273(00)80758-8 10402198

[brb32127-bib-0021] Ezzati, A., Katz, M. J., Zammit, A. R., Lipton, M. L., Zimmerman, M. E., Sliwinski, M. J., & Lipton, R. B. (2016). Differential association of left and right hippocampal volumes with verbal episodic and spatial memory in older adults. Neuropsychologia, 93, 380–385. 10.1016/j.neuropsychologia.2016.08.016 27542320 PMC5154822

[brb32127-bib-0022] Freitas, S., Simões, M. R., Alves, L., & Santana, I. (2011). Montreal Cognitive Assessment (MoCA): Normative study for the Portuguese population. Journal of Clinical and Experimental Neuropsychology, 33, 989–996. 10.1080/13803395.2011.589374 22082082

[brb32127-bib-0023] Gaffan, D. (1994). Scene‐specific memory for objects: A model of episodic memory impairment in monkeys with fornix transection. Journal of Cognitive Neuroscience, 6, 305–320. 10.1162/jocn.1994.6.4.305 23961727

[brb32127-bib-0024] Grady, C., McIntosh, A., Horwitz, B., Maisog, J., Ungerleider, L., Mentis, M., Pietrini, P., Schapiro, M., & Haxby, J. (1995). Age‐related reductions in human recognition memory due to impaired encoding. Science, 269, 218–221. 10.1126/science.7618082 7618082

[brb32127-bib-0025] Graham, K. S., Barense, M. D., & Lee, A. C. H. (2010). Going beyond LTM in the MTL: A synthesis of neuropsychological and neuroimaging findings on the role of the medial temporal lobe in memory and perception. Neuropsychologia, 48, 831–853. 10.1016/j.neuropsychologia.2010.01.001 20074580

[brb32127-bib-0026] Grill‐Spector, K. (2003). The neural basis of object perception. Current Opinion in Neurobiology, 13, 159–166. 10.1016/S0959-4388(03)00040-0 12744968

[brb32127-bib-0027] Grill‐Spector, K., Knouf, N., & Kanwisher, N. (2004). The fusiform face area subserves face perception, not generic within‐category identification. Nature Neuroscience, 7, 555–562. 10.1038/nn1224 15077112

[brb32127-bib-0028] Grill‐Spector, K., Weiner, K. S., Kay, K., & Gomez, J. (2017). The functional neuroanatomy of human face perception. Annual Review of Vision Science, 3, 167–196. 10.1146/annurev-vision-102016-061214 PMC6345578 28715955

[brb32127-bib-0029] Grossi, D., Soricelli, A., Ponari, M., Salvatore, E., Quarantelli, M., Prinster, A., & Trojano, L. (2014). Structural connectivity in a single case of progressive prosopagnosia: The role of the right inferior longitudinal fasciculus. Cortex, 56, 111–120. 10.1016/j.cortex.2012.09.010 23099263

[brb32127-bib-0030] Gschwind, M., Pourtois, G., Schwartz, S., Van De Ville, D., & Vuilleumier, P. (2012). White‐matter connectivity between face‐responsive regions in the human brain. Cerebral Cortex, 22, 1564–1576. 10.1093/cercor/bhr226 21893680

[brb32127-bib-0031] Gutchess, A. H., Welsh, R. C., Hedden, T., Bangert, A., Minear, M., Liu, L. L., & Park, D. C. (2005). Aging and the neural correlates of successful picture encoding: Frontal activations compensate for decreased medial‐temporal activity. Journal of Cognitive Neuroscience, 17, 84–96. 10.1162/0898929052880048 15701241

[brb32127-bib-0032] Habak, C., Wilkinson, F., & Wilson, H. R. (2008). Aging disrupts the neural transformations that link facial identity across views. Vision Research, 48, 9–15. 10.1016/j.visres.2007.10.007 18054981 PMC4828250

[brb32127-bib-0033] Hayes, A. F. (2012). PROCESS: A versatile computational tool for observed variable moderation, mediation, and conditional process modeling. Manuscript Submitted for Publication.

[brb32127-bib-0034] Heath, F., Hurley, S. A., Johansen‐Berg, H., & Sampaio‐Baptista, C. (2018). Advances in noninvasive myelin imaging. Developmental Neurobiology, 78, 136–151. 10.1002/dneu.22552 29082667 PMC5813152

[brb32127-bib-0035] Herbet, G., Zemmoura, I., & Duffau, H. (2018). Functional anatomy of the inferior longitudinal fasciculus: From historical reports to current hypotheses. Frontiers in Neuroanatomy, 12, 77. 10.3389/fnana.2018.00077 30283306 PMC6156142

[brb32127-bib-0036] Hodgetts, C. J., Postans, M., Shine, J. P., Jones, D. K., Lawrence, A. D., & Graham, K. S. (2015). Dissociable roles of the inferior longitudinal fasciculus and fornix in face and place perception. eLife, 4, e07902. 10.7554/eLife.07902 PMC4586481 26319355

[brb32127-bib-0037] Hofstetter, S., Tavor, I., Moryosef, S. T., & Assaf, Y. (2013). Short‐term learning induces white matter plasticity in the fornix. Journal of Neuroscience, 33, 12844–12850. 10.1523/JNEUROSCI.4520-12.2013 23904619 PMC6618548

[brb32127-bib-0038] Irfanoglu, M. O., Walker, L., Sarlls, J., Marenco, S., & Pierpaoli, C. (2012). Effects of image distortions originating from susceptibility variations and concomitant fields on diffusion MRI tractography results. NeuroImage, 61, 275–288. 10.1016/j.neuroimage.2012.02.054 22401760 PMC3653420

[brb32127-bib-0039] Jeurissen, B., Leemans, A., Jones, D. K., Tournier, J. D., & Sijbers, J. (2011). Probabilistic fiber tracking using the residual bootstrap with constrained spherical deconvolution. Human Brain Mapping, 32, 461–479. 10.1002/hbm.21032 21319270 PMC6869960

[brb32127-bib-0040] Jiang, X., Petok, J. R., Howard, D. V., & Howard Jr., J. H. (2017). Individual differences in cognitive function in older adults predicted by neuronal selectivity at corresponding brain regions. Frontiers in Aging Neuroscience, 9, 103. 10.3389/fnagi.2017.00103 28458636 PMC5394166

[brb32127-bib-0041] Jones, D. K., Knösche, T. R., & Turner, R. (2013). White matter integrity, fiber count, and other fallacies: The do’s and don’ts of diffusion MRI. NeuroImage, 73, 239–254. 10.1016/j.neuroimage.2012.06.081 22846632

[brb32127-bib-0042] Koen, J. D., Hauck, N., & Rugg, M. D. (2019). The Relationship between age, neural differentiation, and memory performance. Journal of Neuroscience, 39, 149–162. 10.1523/JNEUROSCI 30389841 PMC6325265

[brb32127-bib-0043] Koen, J. D., & Rugg, M. D. (2019). Neural dedifferentiation in the aging brain. Trends in Cognitive Sciences, 23, 547–559. 10.1016/j.tics.2019.04.012 31174975 PMC6635135

[brb32127-bib-0044] Konar, Y., Bennett, P. J., & Sekuler, A. B. (2013). Effects of aging on face identification and holistic face processing. Vision Research, 88, 38–46. 10.1016/j.visres.2013.06.003 23806271

[brb32127-bib-0045] Lee, A. C. H. (2006). Differentiating the Roles of the hippocampus and perirhinal cortex in processes beyond long‐term declarative memory: A double dissociation in Dementia. Journal of Neuroscience, 26(19), 5198–5203. 10.1523/JNEUROSCI.3157-05.2006 16687511 PMC6674247

[brb32127-bib-0046] Lee, A. C. H., Buckley, M. J., Pegman, S. J., Spiers, H., Scahill, V. L., Gaffan, D., Bussey, T. J., Davies, R. R., Kapur, N., Hodges, J. R., & Graham, K. S. (2005). Specialization in the medial temporal lobe for processing of objects and scenes. Hippocampus, 15, 782–797. 10.1002/hipo.20101 16010661

[brb32127-bib-0047] Lee, A. C. H., Yeung, L.‐K., & Barense, M. D. (2012). The hippocampus and visual perception. Frontiers in Human Neuroscience, 6, 91. 10.3389/fnhum.2012.00091 22529794 PMC3328126

[brb32127-bib-0048] Lee, Y., Grady, C. L., Habak, C., Wilson, H. R., & Moscovitch, M. (2011). Face processing changes in normal aging revealed by fMRI adaptation. Journal of Cognitive Neuroscience, 23, 3433–3447. 10.1162/jocn_a_00026 21452937

[brb32127-bib-0049] Leemans, A., Jeurissen, B., Sijbers, J., & Jones, D. K. (2009). ExploreDTI: A graphical toolbox for processing, analyzing, and visualizing diffusion MR data. 17th Annual Meeting of the International Society for Magnetic Resonance in Medicine: (pp3537).Hawaii, USA.

[brb32127-bib-0050] Leemans, A., & Jones, D. K. (2009). The B‐matrix must be rotated when correcting for subject motion in DTI data. Magnetic Resonance in Medicine, 61, 1336–1349. 10.1002/mrm.21890 19319973

[brb32127-bib-0051] Lindenberger, U., von Oertzen, T., Ghisletta, P., & Hertzog, C. (2011). Cross‐sectional age variance extraction: What's change got to do with it? Psychology and Aging, 26, 34–47. 10.1037/a0020525 21417539

[brb32127-bib-0052] Metzler‐Baddeley, C., Jones, D. K., Belaroussi, B., Aggleton, J. P., & O’Sullivan, M. J. (2011). Frontotemporal connections in episodic memory and aging: A diffusion MRI tractography study. Journal of Neuroscience, 31, 13236–13245. 10.1523/JNEUROSCI.2317-11.2011 21917806 PMC6623273

[brb32127-bib-0053] Mielke, M. M., Okonkwo, O. C., Oishi, K., Mori, S., Tighe, S., Miller, M. I., Ceritoglu, C., Brown, T., Albert, M., & Lyketsos, C. G. (2012). Fornix integrity and hippocampal volume predict memory decline and progression to Alzheimer’s disease. Alzheimer’s and Dementia, 8, 105–113. 10.1016/j.jalz.2011.05.2416 PMC3305232 22404852

[brb32127-bib-0054] Mundy, M. E., Downing, P. E., Dwyer, D. M., Honey, R. C., & Graham, K. S. (2013). A critical role for the hippocampus and perirhinal cortex in perceptual learning of scenes and faces: Complementary findings from amnesia and fMRI. Journal of Neuroscience, 33, 10490–10502. 10.1523/JNEUROSCI.2958-12.2013 23785161 PMC3722491

[brb32127-bib-0055] Nasreddine, Z. S., Phillips, N. A., BÃ©dirian, V. Ã., Charbonneau, S., Whitehead, V., Collin, I., Cummings, J. L., & Chertkow, H. (2005). The Montreal Cognitive Assessment, MoCA: A brief screening tool for mild cognitive impairment. Journal of the American Geriatrics Society, 53, 695–699. 10.1111/j.1532-5415.2005.53221.x 15817019

[brb32127-bib-0056] Nelson, A. J. D., Kinnavane, L., Amin, E., O'Mara, S. M., & Aggleton, J. P. (2020). Deconstructing the direct reciprocal hippocampal‐anterior thalamic pathways for spatial learning. Journal of Neuroscience, 40, 6978–6990. 10.1523/JNEUROSCI.0874-20.2020 32753513 PMC7470921

[brb32127-bib-0057] O’Keefe, J., Burgess, N., Donnett, J. G., Jeffery, K. J., & Maguire, E. A. (1998). Place cells, navigational accuracy, and the human hippocampus. Philosophical Transactions of the Royal Society B: Biological Sciences, 353, 1333–1340. 10.1098/rstb.1998.0287 PMC1692339 9770226

[brb32127-bib-0058] O’Neil, E. B., Barkley, V. A., & Köhler, S. (2013). Representational demands modulate involvement of perirhinal cortex in face processing. Hippocampus, 23, 592–605. 10.1002/hipo.22117 23460411

[brb32127-bib-0059] O’Neil, E. B., Hutchison, R. M., McLean, D. A., & Köhler, S. (2014). Resting‐state fMRI reveals functional connectivity between face‐selective perirhinal cortex and the fusiform face area related to face inversion. NeuroImage, 92, 349–355. 10.1016/j.neuroimage.2014.02.005 24531049

[brb32127-bib-0060] Oliva, A., & Torralba, A. (2001). Modeling the shape of the scene: A holistic representation of the spatial envelope. International Journal of Computer Vision, 42, 145–175. 10.1023/A:1011139631724

[brb32127-bib-0061] O'Mara, S. M., & Aggleton, J. P. (2019). Space and memory (Far) beyond the hippocampus: Many subcortical structures also support cognitive mapping and mnemonic processing. Frontiers in Neural Circuits, 13, 52. 10.3389/fncir.2019.00052 31447653 PMC6692652

[brb32127-bib-0062] Owen, A. M., McMillan, K. M., Laird, A. R., & Bullmore, E. (2005). N‐back working memory paradigm: A meta‐analysis of normative functional neuroimaging studies. Human Brain Mapping, 25, 46–59. 10.1002/hbm.20131 15846822 PMC6871745

[brb32127-bib-0063] Park, D. C., Polk, T. A., Park, R., Minear, M., Savage, A., & Smith, M. R. (2004). Aging reduces neural specialization in ventral visual cortex. Proceedings of the National Academy of Sciences of the United States of America, 101, 13091–13095. 10.1073/pnas.0405148101 15322270 PMC516469

[brb32127-bib-0064] Park, D. C., Welsh, R. C., Marshuetz, C., Gutchess, A. H., Mikels, J., Polk, T. A., Noll, D. C., & Taylor, S. F. (2003). Working memory for complex scenes: Age differences in frontal and hippocampal activations. Journal of Cognitive Neuroscience, 15, 1122–1234. 10.1162/089892903322598094 14709231

[brb32127-bib-0065] Park, J., Carp, J., Hebrank, A., Park, D. C., & Thad, A. P. (2010). Neural specificity predicts fluid processing ability in older adults. Journal of Neuroscience, 30, 9253–9259. 10.1523/JNEUROSCI.0853-10.2010 20610760 PMC2913723

[brb32127-bib-0066] Park, J., Carp, J., Kennedy, K. M., Rodrigue, K. M., Bischof, G. N., Huang, C.‐M., Rieck, J. R., Polk, T. A., & Park, D. C. (2012). Neural broadening or neural attenuation? Investigating age‐related dedifferentiation in the face network in a large lifespan sample. Journal of Neuroscience, 32, 2154–21548. 10.1523/JNEUROSCI.4494-11.2012 22323727 PMC3361757

[brb32127-bib-0067] Patenaude, B., Smith, S. M., Kennedy, D. N., & Jenkinson, M. (2011). A Bayesian model of shape and appearance for subcortical brain segmentation. NeuroImage, 56, 907–922. 10.1016/j.neuroimage.2011.02.046 21352927 PMC3417233

[brb32127-bib-0068] Payer, D., Marshuetz, C., Sutton, B., Hebrank, A., Welsh, R. C., & Park, D. C. (2006). Decreased neural specialization in old adults on a working memory task. NeuroReport, 17, 487–491. 10.1097/01.wnr.0000209005.40481.31 16543812

[brb32127-bib-0069] Pitcher, D., Walsh, V., & Duchaine, B. (2011). The role of the occipital face area in the cortical face perception network. Experimental Brain Research, 209, 481–493. 10.1007/s00221-011-2579-1 21318346

[brb32127-bib-0070] Postans, M., Hodgetts, C. J., Mundy, M. E., Jones, D. K., Lawrence, A. D., & Graham, K. S. (2014). Interindividual variation in fornix microstructure and macrostructure is related to visual discrimination accuracy for scenes but not faces. Journal of Neuroscience, 34, 12121–12126. 10.1523/jneurosci.0026-14.2014 25186756 PMC4152609

[brb32127-bib-0071] Pyles, J. A., Verstynen, T. D., Schneider, W., & Tarr, M. J. (2013). Explicating the face perception network with white matter connectivity. PLoS One, 8, e61611. 10.1371/journal.pone.0061611 23630602 PMC3632522

[brb32127-bib-0072] Qian, H., Wang, Z., Yan, L., & Gao, X. (2017). Aging strikes the self‐face advantage in featural processing. Experimental Aging Research, 43, 379–390. 10.1080/0361073X.2017.1333834 28718751

[brb32127-bib-0073] Rebola, J., & Castelo‐Branco, M. (2014). Visual areas PPA and pSTS diverge from other processing modules during perceptual closure: Functional dichotomies within category selective networks. Neuropsychologia, 61, 135–142. 10.1016/j.neuropsychologia.2014.06.010 24949555

[brb32127-bib-0074] Rieck, J. R., Rodrigue, K. M., Park, D. C., & Kennedy, K. M. (2020). White matter microstructure predicts focal and broad functional brain dedifferentiation in normal aging. Journal of Cognitive Neuroscience, 32, 1536–1549. 10.1162/jocn_a_01562 32286134 PMC8098673

[brb32127-bib-0075] Rivolta, D., Palermo, R., & Schmalzl, L. (2013). What is overt and what is covert in congenital prosopagnosia? Neuropsychology Review, 23, 111–116. 10.1007/s11065-012-9223-0 23152134

[brb32127-bib-0076] Rousselet, G. A., Husk, J. S., Pernet, C. R., Gaspar, C. M., Bennett, P. J., & Sekuler, A. B. (2009). Age‐related delay in information accrual for faces: Evidence from a parametric, single‐trial EEG approach. BMC Neuroscience, 10, 114. 10.1186/1471-2202-10-114 19740414 PMC2746225

[brb32127-bib-0077] Scherf, K. S., Thomas, C., Doyle, J., & Behrmann, M. (2014). Emerging structure‐function relations in the developing face processing system. Cerebral Cortex, 24, 2964–2980. 10.1093/cercor/bht152 23765156 PMC4200039

[brb32127-bib-0078] Schiltz, C., & Rossion, B. (2006). Faces are represented holistically in the human occipito‐temporal cortex. NeuroImage, 32, 385–394. 10.1016/j.neuroimage.2006.05.037 16870475

[brb32127-bib-0079] Smith, S. M., De Stefano, N., Jenkinson, M., & Matthews, P. M. (2001). Normalized accurate measurement of longitudinal brain change. Journal of Computer Assisted Tomography, 25, 466–475. 10.1097/00004728-200105000-00022 11351200

[brb32127-bib-0080] Soares, J. M., Marques, P., Alves, V., & Sousa, N. (2013). A hitchhiker’s guide to diffusion tensor imaging. Frontiers in Neuroscience, 7, 31. 10.3389/fnins.2013.00031 23486659 PMC3594764

[brb32127-bib-0081] Srokova, S., Hill, P. F., Koen, J. D., King, D. R., & Rugg, M. D. (2020). Neural differentiation is moderated by age in scene‐selective, but not face‐selective, cortical regions. eNeuro, 7(3), ENEURO.0142‐20.2020. 10.1523/ENEURO.0142-20.2020 PMC7242814 32341120

[brb32127-bib-0082] Thoma, P., Soria Bauser, D., & Suchan, B. (2013). BESST (Bochum Emotional Stimulus Set) – A pilot validation study of a stimulus set containing emotional bodies and faces from frontal and averted views. Psychiatry Research, 209, 98–109. 10.1016/j.psychres.2012.11.012 23219103

[brb32127-bib-0083] Thomas, C., Avidan, G., Humphreys, K., Jung, K. J., Gao, F., & Behrmann, M. (2009). Reduced structural connectivity in ventral visual cortex in congenital prosopagnosia. Nature Neuroscience, 12, 29–31. 10.1038/nn.2224 19029889 PMC5989137

[brb32127-bib-0084] Tusa, R. J., & Ungerleider, L. G. (1985). The inferior longitudinal fasciculus: A reexamination in humans and monkeys. Annals of Neurology, 18, 583–591. 10.1002/ana.410180512 4073852

[brb32127-bib-0085] Ungerleider, L. G., & Haxby, J. V. (1994). “What” and “where” in the human brain. Current Opinion in Neurobiology, 4, 157–165. 10.1016/0959-4388(94)90066-3 8038571

[brb32127-bib-0086] Wakana, S., Caprihan, A., Panzenboeck, M. M., Fallon, J. H., Perry, M., Gollub, R. L., Hua, K., Zhang, J., Jiang, H., Dubey, P., Blitz, A., van Zijl, P., & Mori, S. (2007). Reproducibility of quantitative tractography methods applied to cerebral white matter. NeuroImage, 36, 630–644. 10.1016/j.neuroimage.2007.02.049 17481925 PMC2350213

[brb32127-bib-0087] Wickens, T. D. (2010). Elementary signal detection theory. Elementary Signal Detection Theory. 10.1093/acprof:oso/9780195092509.001.0001

[brb32127-bib-0088] Willenbockel, V., Sadr, J., Fiset, D., Horne, G. O., Gosselin, F., & Tanaka, J. W. (2010). Controlling low‐level image properties: The SHINE toolbox. Behavior Research Methods, 42, 671–684. 10.3758/BRM.42.3.671 20805589

[brb32127-bib-0089] Wilson, C. R. E., Baxter, M. G., Easton, A., & Gaffan, D. (2008). Addition of fornix transection to frontal‐temporal disconnection increases the impairment in object‐in‐place memory in macaque monkeys. European Journal of Neuroscience, 27, 1814–1822. 10.1111/j.1460-9568.2008.06140.x PMC2327205 18380673

[brb32127-bib-0090] Zebrowitz, L., Ward, N., Boshyan, J., Gutchess, A., & Hadjikhani, N. (2016). Dedifferentiated face processing in older adults is linked to lower resting state metabolic activity in fusiform face area. Brain Research, 1644, 22–31. 10.1016/j.brainres.2016.05.007 27163722 PMC4903926

[brb32127-bib-0091] Zhuang, L., Sachdev, P. S., Trollor, J. N., Reppermund, S., Kochan, N. A., Brodaty, H., & Wen, W. (2013). Microstructural white matter changes, not hippocampal atrophy, detect early amnestic mild cognitive impairment. PLoS One, 8, e58887. 10.1371/journal.pone.0058887 23516569 PMC3597581

