# Supplementary material for: Structural impairments in hippocampal and occipitotemporal networks specifically contribute to decline in place and face category processing but not to other visual object categories in healthy aging
Source: Brain Behav. 2021 Jun 29;11(8):e02127. doi: 10.1002/brb3.2127 (PMC8413757; doi:10.1002/brb3.2127)
Supplement: Supplementary file 1 — Fig S1 [file BRB3-11-e02127-s003.pdf]

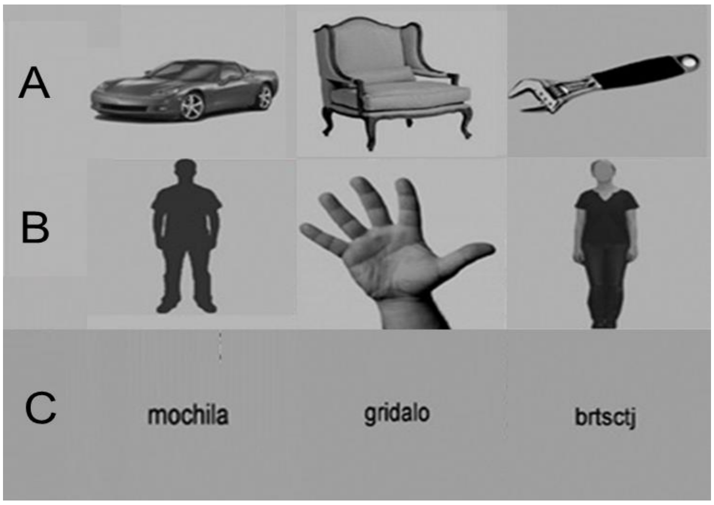

Figure S1. Depicts examples of control stimulus categories used for this study. (A) The object category was composed of cars, chairs and tools (B) body categories were represented by faceless bodies, hands and feet and body shape silhouettes and (C) verbal category was composed of real words, pseudowords and nonwords. Please note that the faceless bodies images were taken from the Bochum Emotional Stimulus Set database (Thoma et al., 2013), and hands and feet were selected from publicly available images that were obtained online. Body shape silhouettes images were generated with a customized code in MATLAB R2014a (MathWorks, Natick, USA). The verbal material was provided as a courtesy from the database of the Universidade Católica Portuguesa.
