# Supplementary material for: Structural impairments in hippocampal and occipitotemporal networks specifically contribute to decline in place and face category processing but not to other visual object categories in healthy aging
Source: Brain Behav. 2021 Jun 29;11(8):e02127. doi: 10.1002/brb3.2127 (PMC8413757; doi:10.1002/brb3.2127)
Supplement: Supplementary file 2 — Fig S2 [file BRB3-11-e02127-s001.pdf]

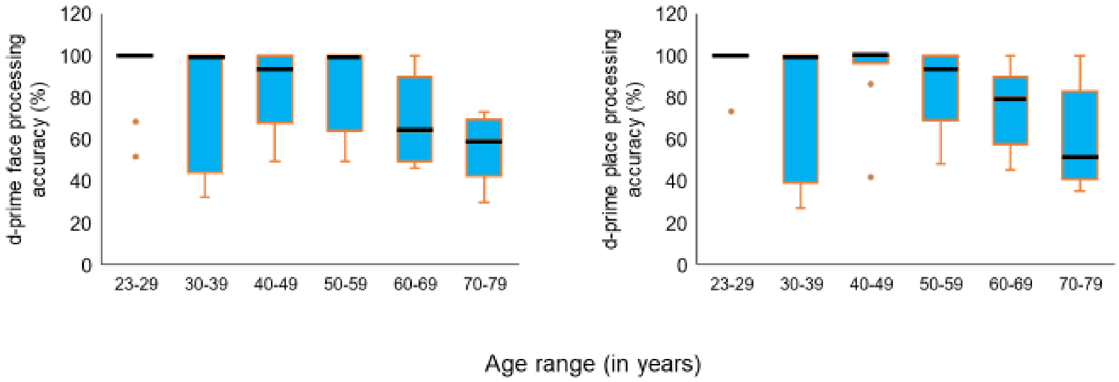

Figure S2. Depicts boxplots of cognitive performance for faces and place categories by age range. Please note the thick black lines represent the performance median.
