# Supplementary material for: Structural impairments in hippocampal and occipitotemporal networks specifically contribute to decline in place and face category processing but not to other visual object categories in healthy aging
Source: Brain Behav. 2021 Jun 29;11(8):e02127. doi: 10.1002/brb3.2127 (PMC8413757; doi:10.1002/brb3.2127)
Supplement: Supplementary file 3 — Table S1 [file BRB3-11-e02127-s002.docx]

Table S1. Depicts the outcome of all mediation analysis testing indirect effects of micro-and macrostructural variables on the direct effects of age on cognitive performances for visual object categories.

|  | Model 1 | Model 2 | Model 3 | Model 4 | Model 5 |
| --- | --- | --- | --- | --- | --- |
| Mediators | IV = age  DV= faces | IV = age  DV= places | IV = age  DV = objects | IV = age  DV = bodies | IV = age  DV = verbal |
| *Microstructure*  *(FA)* | a: t= - 6.3***  b: t= 1.2  c´: t = -1.2  a*b: b= -0.014  (-0.04, 0.008) | **a: t= - 6.3*****  **b: t= 2.5****  **c´: t = 0.04**  **a*b: b= -0.028**  **(-0.054, -0.006)** | a: t= - 6.3***  b: t= -0.002  c´: t = -2.7**  a*b: b = 0  (-0.027, 0.02) | a: t= - 6.3***  b: t= 1.76  c´: t = -0.6  a*b: b= -0.02  (-0.04, 0.005) | a: t= - 6.3***  b: t= 1  c´: t = -0.9  a*b: b= -0.01  (-0.04, 0.009) |
| Fornix |  |  |  |  |  |
| R ILF | **a: t= - 2.5****  **b: t= 2***  **c´: t = -1.8**  **a*b: b= -0.009**  **(-0.03, -0.0009**) | a: t= - 2.5**  b: t= 1.1  c´: t = -1.56  a*b: b= -0.005  (-0.02, 0.002) | a: t= - 2.5**  b: t= 1.5  c´: t = -2.97**  a*b: b = -0.006  (-0.022, 0.0016) | a: t= - 2.5**  b: t= 1.3  c´: t = -1.7  a*b: b= -0.006  (-0.02, 0.002) | a: t= - 2.5**  b: t= 0.5  c´: t = -1.76  a*b: b= -0.002  (-0.01, 0.006) |
| L ILF | a: t= - 1.45  b: t= 0.32  c´: t = -2.5**  a*b: b= -0.0009  (-0.009, 0.003) | a: t= - 1.45  b: t= 0.23  c´: t = -1.9  a*b: b= -0.0006  (-0.009, 0.004) | a: t= - 1.45  b: t= -0.4  c´: t = -3.6***  a*b: b = 0.001  (-0.004, 0.006) | a: t= - 1.45  b: t= -0.4  c´: t = -2.3*  a*b: b= 0.001  (-0.005, 0.009) | a: t= - 1.45  b: t= -1.3  c´: t = -2.3*  a*b: b= 0.003  (-0.003, 0.01) |
| R ATR | a: t= - 3.1**  b: t= -0.55  c´: t = -2.63**  a*b: b= 0.0032  (-0.008, 0.013) | a: t= -3.1**  b: t= -0.6  c´: t = -2.1*  a*b: b= 0.0036  (-0.009, 0.014) | a: t= - 3.1**  b: t= -0.18  c´: t = -3.4**  a*b: b = 0.001  (-0.01, 0.0098) | a: t= - 3.1**  b: t= 0.48  c´: t = -1.9  a*b: b= 0.0028  (-0.01, 0.007) | a: t= - 3.1**  b: t= -1.7  c´: t = -2.6**  a*b: b= 0.01  (-0.0002, 0.02) |
| L ATR | a: t= - 2.7**  b: t= 0.33  c´: t = -2.35*  a*b: b= -0.0016  (-0.011, 0.007) | a: t= -2.7**  b: t= -0.8  c´: t = -2.2*  a*b: b= 0.004  (-0.004, 0.014) | a: t= - 2.7**  b: t= 0.3  c´: t = -3.3**  a*b: b = -0.001  (-0.01, 0.008) | a: t= - 2.7**  b: t= -0.36  c´: t = -2.2*  a*b: b= 0.0018  (-0.008, 0.01) | a: t= - 2.7**  b: t= -1.8  c´: t = -2.6**  a*b: b= 0.0098  (-0.0003, 0.02) |

| *Macrostructure*  *(Volume)* |  |  |  |  |  |
| --- | --- | --- | --- | --- | --- |
| R HC | a: t= - 2.6**  b: t= 2.2*  c´: t = -1.8  a*b: b= -0.01  (-0.02, 0.000) | **a: t= -2.6****  **b: t= 2.6****  **c´: t = -1.1**  **a*b: b= -0.01**  **(-0.02, -0.0006)** | a: t= - 2.6**  b: t= 0.32  c´: t = -3.2**  a*b: b = -0.001  (-0.01, 0.008) | a: t= - 2.6**  b: t= 1.3  c´: t = -1.7  a*b: b= -0.006  (-0.02, 0.002) | a: t= - 2.6**  b: t= 1.6  c´: t = -1.4  a*b: b= -0.008  (-0.02, 0.0013) |
| L HC | a: t= - 2.3*  b: t= 0.8  c´: t = -2.05*  a*b: b= -0.0035  (-0.015, 0.0085) | a: t= -2.3*  b: t= 0.32  c´: t = -1.57  a*b: b= -0.0014  (-0.01, 0.012) | a: t= - 2.3*  b: t= -0.27  c´: t = -3.3**  a*b: b = -0.001  (-0.008, 0.014) | a: t= - 2.3*  b: t= 0.57  c´: t = -2*  a*b: b= -0.0026  (-0.01, 0.007) | a: t= - 2.3*  b: t= 1.06  c´: t = -1.4  a*b: b= -0.005  (-0.017, 0.005) |

The scores represent the results for all paths of the models (a, b and c´) and the significance of the indirect effects (a*b) (see Figure 1). Significant mediation models are highlighted in bold. 95% confidence interval in brackets were based on bootstrapping with 5000 replacements. *p≤ 0.05; **p≤ 0.01, ***p ≤0.001. IV = independent variable, DV = dependent variable. b = unstandardized coefficients
